# Validation of the North America expert consensus statement on reporting CT findings for COVID-19 in individuals with lung cancer

**DOI:** 10.1590/1414-431X2022e12376

**Published:** 2023-01-09

**Authors:** D. Peixoto, Y.C.S. Neves, G. Generoso, B.M.C. Loureiro, J.P.B. Callia, V.M. Anastácio, J.L. Alves, E.M. Oshiro, L.R. Lima, M.V.Y. Sawamura, R.V. Auad, M.S. Bittencourt, E. Abdala, K.Y. Ibrahim

**Affiliations:** 1 Instituto do Câncer do Estado de São Paulo, Faculdade de Medicina, Universidade de São Paulo, São Paulo, SP, Brasil; 2 Centro de Pesquisa Clínica e Epidemiológica, Hospital Universitário, Universidade de São Paulo, São Paulo, SP, Brasil

**Keywords:** Lung, Patients, Cancer, Radiological Society of North America, Computed tomography, RT-PCR

## Abstract

The aim of our study was to validate the use of the standardized Radiological Society of North America (RSNA) reporting system in individuals with known lung cancer who presented to the emergency department with suspected COVID-19. We included patients aged 18 years or older from the Cancer Institute of the State of São Paulo (ICESP) with a confirmed diagnosis of lung cancer, admitted to the emergency department and undergoing chest computed tomography (CT) for suspicion of COVID-19. Comparison between SARS-CoV2 RT-PCR across RSNA categories was performed in all patients and further stratified by diagnosis of lung cancer progression. Among 58 individuals included in the analysis (65±9 years, 43% men), 20 had positive RT-PCR. Less than a half (43%) had no new lung findings in the CT. Positive RT-PCR was present in 75% of those with typical findings according to RSNA and in only 9% when these findings were classified as atypical or negative (P<0.001). Diagnostic accuracy was even higher when stratified by the presence or absence of progressive disease (PD). Extent of pulmonary inflammatory changes was strongly associated with higher mortality, reaching a lethality of 83% in patients with >25% of lung involvement and 100% when there was >50% of lung involvement. The lung involvement score was also highly predictive of prognosis in this population as was reported for non-lung cancer individuals. Collectively, our results demonstrated that diagnostic and prognostic values of chest CT findings in COVID-19 are robust to the presence of lung abnormalities related to lung cancer.

## Introduction

Coronavirus disease 2019 (COVID-19) has rapidly spread throughout the world (1). Although most cases are mild, it may also be associated with severe illness, particularly in high-risk individuals such as cancer patients (2). Lung cancer is a unique condition in which several risk factors for COVID-19 complications are common, such as older age, smoking habits, and pre-existing cardiopulmonary comorbidities, in addition to cancer treatments (3,4). Additionally, lung cancer has the highest morbidity and mortality among individuals with any type of cancer. Some reports highlight the high proportion of patients with lung cancer and confirmed COVID-19 who develop severe adverse outcomes (1,4-6).

Pulmonary abnormalities are commonly described in COVID-19 individuals who undergo radiological examinations, particularly in computed tomography (CT) scans (7). Up until the second day, even CTs are unlikely to show abnormalities with a common dissociation between clinical, laboratory, and imaging findings in up to 50% of patients (8). Later in the course of the disease, CT has high sensitivity but limited specificity to characterize COVID-19 (8-10). The most common lung changes are bilateral round ground-glass opacities and consolidation foci with a typical peripheral distribution. Pleural effusion, pleural thickening, and lymphadenopathy have been reported in a minority of patients and thought to be not directly associated to viral infection (11-13).

In order to reduce variability and improve the diagnostic performance of chest CT imaging in COVID-19, the Radiological Society of North America (RSNA) has developed a standardized reporting system (14). This system has been validated as a useful tool in clinical practice with a high accuracy in a setting of epidemic spread, with high pre-test probability of COVID-19 (15). However, individuals with lung cancer already have prevalent or persistent lung abnormalities that might interfere with the performance of the standardized system, and the SARS-CoV-2 infection is unlikely to affect all patients with cancer equally (4). Thus, the diagnostic accuracy of CT in lung cancer patients with suspected COVID-19 is likely more prone to misclassification due to prior disease as well as side effects from chemoradiotherapy.

Thus, in the present study we sought to validate the RSNA standardized reporting system in individuals with known prevalent lung cancer who present to the emergency department with suspected COVID-19.

## Material and Methods

### Ethics approval

This retrospective study was approved by the Institutional Review Board (protocol 1737/20), which granted a waiver for informed consent due to the retrospective nature of the study.

### Study design and participants

This cohort study included adult patients (≥18 years old) from the State of São Paulo Cancer Institute (ICESP), a tertiary care oncological hospital. We included all individuals with a confirmed histopathological diagnosis of lung cancer and suspected COVID-19, defined by the presence of at least one of the following symptoms: fever higher than 37.8°C, cough, sore throat, rhinorrhea, dyspnea, anosmia, dysgeusia, oxygen saturation <93%, or respiratory rate >24 incursions per minute (based on the criteria published by the WHO (16)) who presented to the emergency department of the ICESP, Brazil, from March 23 to May 31, 2020 and underwent CT for suspected COVID-19.

We collected information on symptoms and signs on admission including age, sex, cancer histological type, tumor staging, immunosuppressive regimen treatment, comorbidities, smoking status (current, former, or never smoker), Eastern Cooperative Oncology Group (ECOG) performance status (17), hospital and Intensive Care Unit (ICU) admission, mechanical ventilation, and mortality from medical records.

The histological type was stratified into two groups: i) small cell carcinoma and ii) non-small cell carcinoma, which included: adenocarcinoma, squamous cell carcinoma, and giant cell carcinoma. Tumor staging followed the American Joint Committee on Cancer (AJCC Cancer Staging Manual) (18). Due to the limited sample size, we grouped stages I, II, and III into one major group and stage IV (metastasis) into another group. ECOG score of the last outpatient visit prior to admission was considered and was then stratified into two categories: scores 1 and 2 *vs* scores 3 and 4.

The anticancer therapy was defined as either cytotoxic chemotherapy or all other therapies such as targeted drugs, endocrine therapy, or immunotherapy, administered within 30 days prior to the admission. Radiotherapy or surgery were also analyzed if performed within 30 days prior to admission. We defined palliative care as present if the patient was previously followed by the specialized palliative care team as an outpatient or had received this status prior the COVID-19 investigation.

Other clinical variables (comorbidities such as COPD, diabetes mellitus, and hypertension) were collected from medical records. We stratified the smoking status as current for all those currently smoking or those who stopped smoking up to one month prior to the admission and former for those who stopped smoking more than one month before the onset of symptoms.

Clinical samples for SARS-CoV-2 RT-PCR consisted in naso- and oropharyngeal swabs, from which nucleic acid (RNA) was extracted with an automated method based on magnetic beads (mSample Preparation System RNA, Abbott, USA). Reverse transcription, amplification, and detection were performed following an in-house protocol validated in the Laboratory Division (CAP accredited) comprising an E gene assay as the first-line screening tool, followed by confirmatory testing with an N gene assay, as previously described (19). Endogenous gene RNAseP was used as the internal control of extraction and amplification, as well as positive and negative external controls. Analytical sensitivity was 40 copies/mL and specificity in samples containing other respiratory virus RNAs was 100%. Molecular testing results were obtained through institutional databases. The COVID-19 confirmed case was defined as a positive RT-PCR SARS-CoV-2 from an oral and nasopharyngeal swab or endotracheal aspirate specimen. Asymptomatic patients with RT-PCR collected for other institutional protocols were not included.

### CT acquisition technique

Chest CT was performed in the supine position during inspiratory breath-hold from the apex to the lung bases, on either a 64-slice CT scanner (Philips Brilliance, Philips Healthcare, The Netherlands) or on a 128-slice scanner (Toshiba Aquillion, Japan) with or without intravenous contrast media administration at the physician's discretion. Scanning parameters followed institutional protocols for each machine and patient body type and were collimated at 64×0.625 or 0.6 mm, 120 kVp, 667 max mA or 404 max mA, pitch 1.0 or 1.2, and matrix size 512×512. CT images were reconstructed in the transverse plane with 1.0-mm slice thickness and 1.0-mm increment. All images were stored and accessed through an integrated PACS (Picture Archiving and Communication System, PHILIPS iSite, version 4.1.110.0, USA).

### CT images analysis

CT scans were retrospectively and independently read by two chest radiologists with 6 and 5 years of experience in chest CT analysis. At the time of chest CT interpretation, the readers were only aware of age and gender and thus were blinded to RT-PCR results and other clinical characteristics. Each patient was categorized into one of the four categories proposed by the RSNA Expert Consensus Statement (14): 1 - typical, 2 - indeterminate, 3 - atypical, or 4 - negative. The detailed classification is provided in the Supplementary Table S1. Both readers had access to previous CT images when available at the institution and used them to better categorize the patient into the four categories mentioned.

Opacities previously observed in scans acquired more than 1 month before the current exam and in the same locations were considered not suspicious for COVID-19 (and thus classified as “negative” or “atypical”, as appropriate). Final RSNA category was achieved by consensus. Findings related to the tumor (e.g., direct tumor infiltration, enlarged lymph nodes or malignant pleural implants and effusion) were not considered to be associated to acute inflammatory process. Examiners considered “progressive disease” (PD) if there was at least 20% increase in the sum of diameters of target lesions (and a minimum absolute increase of 5 mm in this sum), following the RECIST 1.1 guidelines (20).

The extent of pulmonary changes was also recorded, considering as a reference (denominator) only viable lung parenchyma and using two visual scores: the first, an overall burden of inflammatory lung changes (ranges of 1 to <25, 25 to <50%, 50 to <75% and 75 to 100%); and a second (and more refined) scale, for which the lungs were divided into three zones (upper zone, above the carina level; lower zone, below the infrapulmonary vein level; middle zone, between the upper and lower zones) and categorized using the same ranges aforementioned, attributing a score of 0-4 for each zone (maximum of 24 if the two lungs were viable; a percentage was then generated). If there was only one viable lung (the other was affected by neoplastic disease or withdrawn due to post-treatment changes), only this was taken into consideration for calculation (e.g., fraction denominator of 12 points, not 24). Final overall extent was reached by consensus; final refined extent (in %) was determined as the average of the percentages of inflammatory changes given by both examiners.

### Statistical analysis

Continuous data are reported as means and standard deviations or median and quartiles as appropriate. Normality was evaluated by visual inspection of histograms. Categorical variables are reported as counts and percentages. Comparisons between groups were performed using *t*-tests and Kruskal Wallis test (for non-normal variables) for continuous variables and Fisher's exact test for categorical variables. Univariate logistic regressions were used to evaluate predictors of all-cause mortality across those with positive and negative RT-PCR. All the analyses were performed using Stata 14 (StataCorp. College Station, TX: StataCorp LP, USA), and a P value <0.05 was considered significant.

## Results

### Baseline characteristics

We included 58 individuals with confirmed lung cancer who underwent CT (65±9 years, 43% male, 28% with metastatic disease), of which 20 patients had positive RT-PCR (34.5%). Twenty-four (42%) patients received intravenous contrast and 55 had a prior CT (of which 84% presented with new pulmonary changes). The ECOG classification was ≥3 in 17 (29%) individuals. Twenty-four (42%) patients received anticancer therapy up to 30 days prior to the admission (Table 1).

**Table 1:** Patient characteristics according to computed tomography (CT) findings and Radiological Society of North America (RSNA) categories (1 to 4).

RSNA	1 Typical n=12	2 Indeterminate n=23	3 Atypical n=12	4 Negative n=11	Total n=58
Age (mean, SD)	67±9	64±7	65±9	64±13	65±9
Male	4 (33%)	11 (48%)	6 (50%)	4 (36%)	25 (43%)
Stage (N=54)					
I, II, and III	4 (36%)	21 (95%)	8 (80%)	6 (55%)	39 (72%)
IV	7 (65%)	1 (5%)	2 (20%)	5 (45%)	15 (28%)
ECOG ≥3	3 (25%)	8 (35%)	5 (42%)	1 (9%)	17 (29%)
Anticancer therapy prior 30 days	2 (17%)	11 (48%)	5 (42%)	6 (55%)	24 (42%)
Comorbidities					
Hypertension	6 (50%)	9 (39%)	8 (67%)	6 (55%)	29 (50%)
Type 2 diabetes	5 (42%)	7 (30%)	7 (58%)	1 (9%)	20 (34%)
COPD	6 (42%)	6 (26%)	4 (33%)	4 (36%)	19 (33%)
Clinical presentation					
Cough	3 (25%)	12 (52%)	8 (67)	7 (64%)	30 (52%)
Fever	6 (50%)	5 (22%)	4 (33%)	2 (18%)	17 (29%)
Dyspnea	2 (17%)	6 (26%)	2 (17%)	6 (55%)	16 (28%)
Worsening base dyspnea	4 (33%)	13 (57%)	5 (42%)	4 (36%)	26 (45%)

ECOG: Eastern Cooperative Oncology Group; COPD: chronic obstructive pulmonary disease.

At admission, less than one third of the patients presented with fever, about a half had a cough, 16 (28%) had dyspnea, and 26 (45%) had worsening baseline dyspnea. There was no difference between individuals with positive or negative RT-PCR tests regarding any symptoms, smoking status, or COPD. The mean performance status was worse in patients with negative RT-PCR (P<0.001) (Table 2).

**Table 2:** Patient characteristics according to RT-PCR results.

Patient characteristics	RT-PCR positive n=20 (34%)	RT-PCR negative n=38 (66%)	P value
Age (mean, SD)	66±9	64±9	0.43
Male	10 (50%)	15 (39%)	0.58
Histopathology			0.17
Small cell carcinoma	2 (10%)	2 (5%)	
Non-small cell carcinoma	18 (90%)	36 (95%)	
Stage (N=54)			0.60
I, II, and III	7 (39%)	8 (22%)	
IV	11 (61%)	28 (78%)	
ECOG ≥3	2 (10%)	15 (39%)	**<0.003**
Anticancer therapy prior 30 days	9 (45%)	15 (39%)	0.78
Radiotherapy prior 30 days	4 (20%)	04 (11%)	0.43
Comorbidities			
COPD	5 (25%)	14 (37%)	0.40
Hypertension	12 (60%)	17 (45%)	0.41
Type 2 diabetes	7 (35%)	13 (34%)	1.00
Smoking			0.33
Current	2 (10%)	10 (26%)	
Former	13 (65%)	18 (47%)	
Clinical presentation			
Cough	8 (40%)	12 (58%)	0.27
Fever	7 (35%)	10 (26%)	0.55
Dyspnea	7 (35%)	9 (24%)	0.37
Worsening base dyspnea	8 (40%)	18 (47%)	0.78
Sat O_2_ initial presentation	94 (88-96)	92 (85-95)	0.61
Respiratory rate (ipm)	19 (18-22)	20 (18-25)	0.44

Comparisons were performed with *t*-test and Fisher's exact test. P values in bold type are statistically significant. ECOG: Eastern Cooperative Oncology Group; COPD: chronic obstructive pulmonary disease; ipm: inspirations per minute.

### Chest CT

The most common findings were typical changes observed in COVID-19. Ground-glass opacities (90%), crazy-paving pattern (70%), and perilobular opacities (60%) were more common in the positive RT-PCR group, whereas areas of consolidation (55%) and progressive disease (53%) were more frequent in the negative RT-PCR group (Table 3). None of the isolated main lung CT changes were independently associated with any clinical presentation or outcome.

**Table 3:** Computed tomography (CT) imaging findings according to SARS-CoV2 RT-PCR result.

CT findings	RT-PCR positive n=20 (34%)	RT-PCR negative n=38 (66%)	P value
Ground-glass opacities	18 (90%)	27 (71%)	0.18
Consolidation	9 (45%)	21 (55%)	0.58
Reversed halo sign	0	2 (5%)	
Perilobular opacities	12 (60%)	9 (24%)	**0.01**
Crazy-paving pattern	14 (70%)	13 (34%)	**0.01**
Unilateral findings	3 (15%)	10 (26%)	0.51
Central (medullary) distribution	1 (5%)	6 (16%)	0.40
Diffuse pattern	8 (40%)	12 (32%)	0.57
Acute respiratory distress syndrome	2 (10%)	2 (5%)	0.60
Pleural effusion	3 (15%)	9 (24%)	0.52
Bronchitis	2 (10%)	9 (24%)	0.30
Tree-in-bud pattern	1 (5%)	10 (26)	0.08
Septal lines	1 (5%)	2 (5%)	1.0
Radiation therapy-related lung injury (acute or chronic)	4 (20%)	2 (5%)	0.17
Alveolar hemorrhage (suspected)	0	2 (5%)	0.54
Lymphangitis	5 (25%)	12 (32%)	0.76
Progressive disease (n=56)	3 (15%)	20 (53%)	**0.01**

Comparisons were performed with Fisher's exact test. P values in bold type are statistically significant.

### CT image analyses

**Figure 1:**
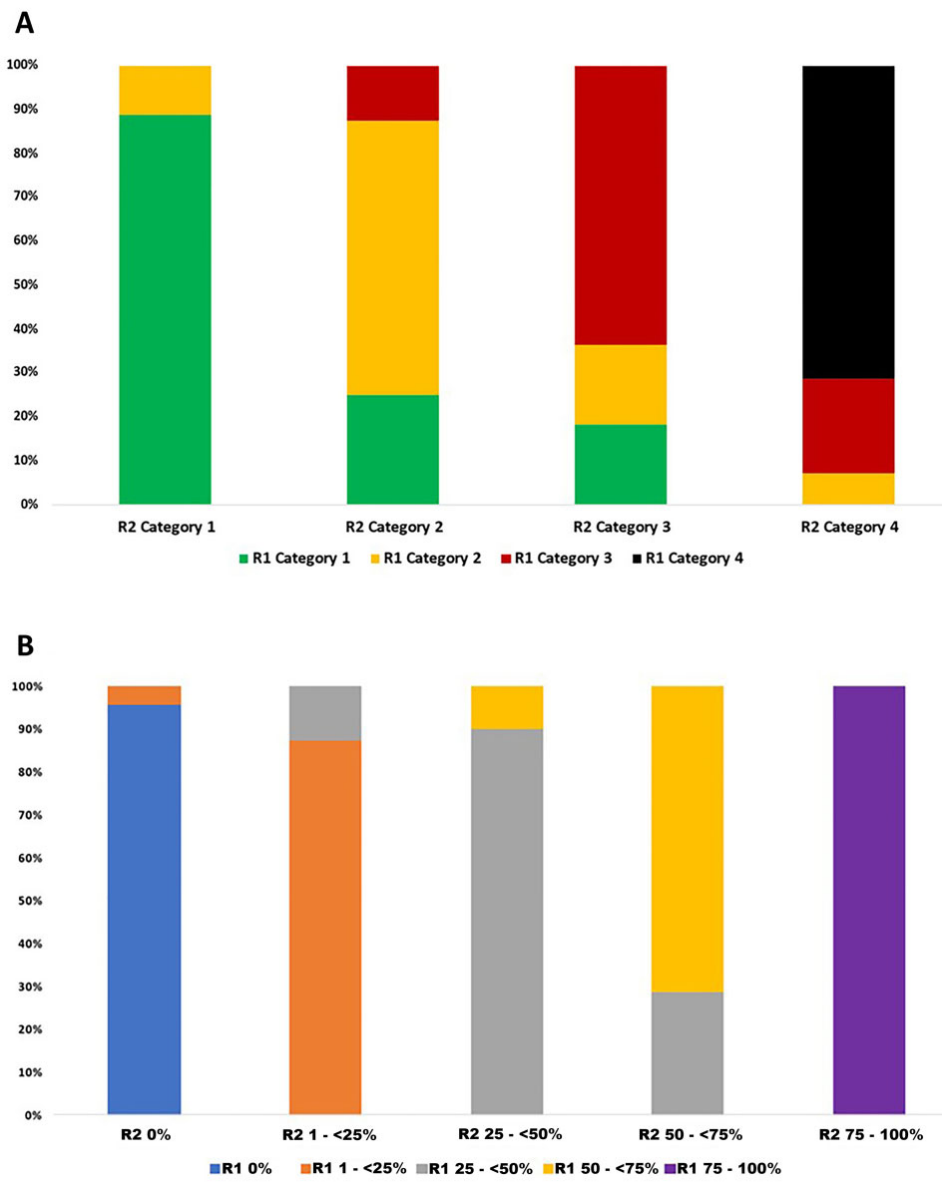
Inter-reader agreement between the two readers for Radiological Society of North America (RSNA) classification (**A**) and for extent of lung involvement (**B**).

The inter-reader agreement between the two readers was κ=0.58 for RSNA category and κ=0.85 for extent of lung involvement (agreement of 69 and 89.7%, respectively; both P<0.001) (Figure 1A and B).

Positive RT-PCR was detected in 75% of patients with typical findings in CT (n=9/12) and in 39% of patients with indeterminate findings (n=9/14), while only in 9% (n=2/21) of patients with atypical or no pulmonary changes (P<0.001)

**Figure 2:**
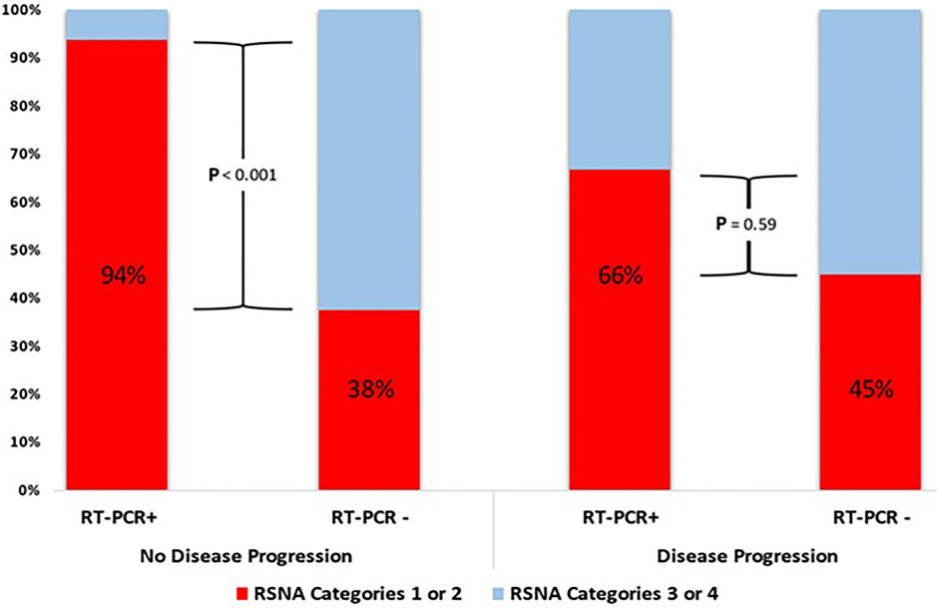
Radiological Society of North America (RSNA) categories according to RT-PCR results and disease progression (Fisher's exact test).

When stratified by disease progression, the association between RSNA categories 1 and 2 and RT-PCR (P=0.001) was observed in those who had a prior CT scan and no progressive disease. However, if there was disease progression, no association was found between RSNA dichotomized classification and RT-PCR result, as the prevalence of positive RT-PCR was low in these subgroups (P=0.59) (Figure 2).

### Extent and mortality

Epidemiological characteristics, as well as oncological diagnosis and treatment, did not differ between survivors and deceased (Table 4). However, tachypnea, hypoxia, lymphopenia, and high plasma levels of C-reactive protein and lactate dehydrogenase were associated with higher mortality (Supplementary Table S2).

**Table 4:** Patient characteristics according to mortality.

Patient characteristics	Survival n=27 (47%)	Deceased n=31 (53%)	P value
Age (mean, SD)	65±6	64±9	0.60
Male	11 (44%)	16 (48%)	0.58
Histopathology			0.61
Small cell carcinoma	1 (4%)	3 (10%)	
Non-small cell carcinoma	26 (96%)	28 (90%)	
Stage (n=54)			**0.07**
I, II, and III	15 (60%)	24 (82%)	
IV	10 (40%)	5 (18%)	
ECOG ≥3	5 (18%)	12 (39%)	0.14
Anticancer therapy prior 30 days	10 (37%)	14 (45%)	0.60
Radiotherapy prior 30 days	5 (18%)	9 (10%)	0.45
Comorbidities			
COPD	9 (33%)	10 (32%)	1.00
Hypertension	14 (45%)	15 (56%)	0.60
Type 2 diabetes	9 (33%)	11 (35%)	1.00
Smoking			0.68
Current	7 (25%)	5 (16%)	
Former	13 (48%)	18 (58%)	
Clinical presentation			
Cough	15 (56%)	15 (48%)	0.61
Fever	9 (33%)	8 (26%)	0.57
Dyspnea	9 (33%)	7 (23%)	0.39
Worsening base dyspnea	9 (33%)	17 (55%)	0.11
Sat O_2_ initial presentation (%) (median, IQR)	95 (92-96)	88 (80-94)	**0.005**
Respiratory rate (ipm) (median, IQR)	19 (18-20)	22 (18-27)	**0.01**

Comparisons were performed with *t*-test or Kruskal Wallis test and Fisher's exact test. P values in bold type are statistically significant. ECOG: Eastern Cooperative Oncology Group; COPD: chronic obstructive pulmonary disease; ipm: inspirations per minute.

**Figure 3:**
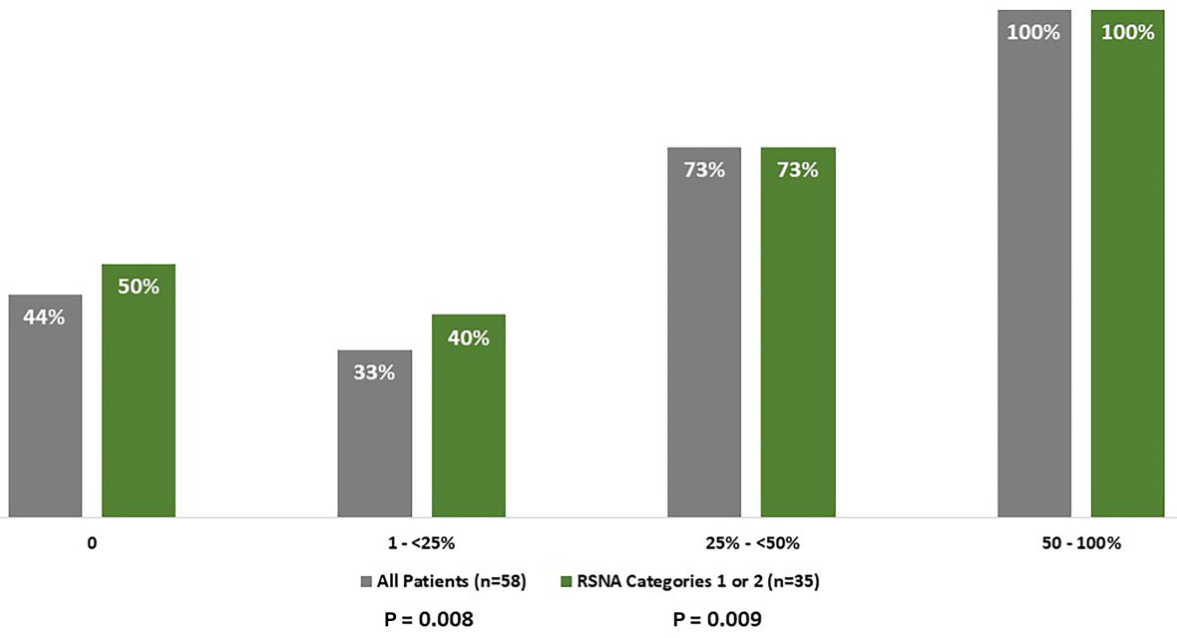
Death rate according to extent of inflammatory changes in all patients and in Radiological Society of North America (RSNA) categories 1 and 2 (Fisher's exact test).

Only 43% of the patients had no lung involvement at hospital admission. As expected, large lung inflammatory changes were directly associated with in-hospital mortality (P=0.008), with a high lethality rate in those with >25% of lung involvement (83%, n=15/18). In a sensitivity analysis with RSNA categories 1 and 2 (n=35) only, we also found a statistically significant association between lung involvement and mortality rate (P=0.009) (Figure 3).

## Discussion

In the present study, we demonstrated that the RSNA standardized reporting system has a robust performance for the identification of positive RT-PCR in individuals with known lung cancer. This was even more pronounced in individuals with no progressive lung cancer. Additionally, we observed a high mortality rate with a direct association between mortality and extent of lung involvement.

The predominant CT findings in COVID-19 are bilateral, peripheral, and basal predominantly ground-glass opacity, consolidation, or both. Opacities often have an extensive geographic distribution (8). The atypical CT findings are only seen in a minority of patients and should raise concern for concomitant bacterial pneumonia or other differential diagnoses (21).

Since early reports from China, chest CT has been considered an adequate tool for the diagnosis of COVID-19 using RT-PCR as a reference standard (22), although other reports suggested only moderate accuracy (23). Due to this limitation, the RSNA proposed a standardized report to improve diagnostic accuracy of chest CT to diagnose COVID-19 (14), and another classification system was also proposed (24). However, this moderate to high diagnostic accuracy came from studies including mostly individuals with no prior lung disease. Since prior disease is expected to interfere with diagnostic accuracy, the results of our study add substantial novel information by demonstrating adequate performance of chest CT in this population. It is interesting to note that the kappa reported in our study was lower than the agreement previously reported (25,26). However, our findings are in line with another study that reported a lower agreement (27). Although this could be explained by several possibilities including training and experience, it is not surprising that the agreement would be lower in more complex patients with baseline lung disease as included in our study.

We demonstrated that the RSNA standardized reporting system had a robust performance for the identification of positive RT-PCR even in individuals with known lung cancer. This was even more pronounced in individuals with no progressive lung cancer disease. Additionally, we observed a high mortality rate with a direct association between mortality and extent of lung involvement. We demonstrated the severity of the association concerning lung cancer and COVID-19. This also makes the CT exam and the RSNA standardized reporting system essential prognostic tools in this group of patients. Our data demonstrated that COVID-19 infection in lung cancer patients is probably more severe than in the general population.

While the use of chest CT to evaluate the prognosis of COVID-19 patients has been reported early in the pandemic (28), the criteria to define higher risk individuals was only proposed later. While some studies reported measures of time-to-improvement in repeated scans (28), most studies focused on quantification of lung involvement as a predictor of outcomes (29-32). In general, results are consistent across studies, though none of the prior reports includes individuals with prior disease. Our findings do corroborate the robustness of lung involvement as a prognostic factor even in individuals with lung cancer.

However, the present study must be viewed within the context of its design. First, the limited sample size, which influences the ability to perform adjustments for several possible confounders. Second, the real-world sensitivity of RT-PCR, including variability of time after symptoms for test collection, might have led to the inclusion of some COVID-19 cases in the negative group (33). Finally, this study was performed in confirmed lung cancer patients followed in a tertiary care center, and the current findings might not be applicable in other scenarios.

To the best of our knowledge, this is the first study in patients with lung cancer to evaluate the performance of the RSNA standardized reporting system. Our results demonstrated that this reporting system is robust even in complex patients with lung cancer. Additionally, our results demonstrated that the lung involvement score is also highly predictive of prognosis in this population as was reported for non-lung cancer individuals. Collectively, our results demonstrated that diagnostic and prognostic value of chest CT findings in COVID-19 are robust in the detection of lung abnormalities related to lung cancer.

## Supplementary Material

Click here to view [pdf].
